# Identification of a potent herbal molecule for the treatment of breast cancer

**DOI:** 10.1186/1471-2407-9-41

**Published:** 2009-01-30

**Authors:** Srinivas Koduru, Srinivasan Sowmyalakshmi, Raj Kumar, Rohini Gomathinayagam, Jürgen Rohr, Chendil Damodaran

**Affiliations:** 1 Department of Clinical Sciences, College of Health Sciences, University of Kentucky, Lexington, KY- 40536, USA; 2 Department of Pharmaceutical Sciences, College of Pharmacy, University of Kentucky, Lexington, KY- 40536, USA

## Abstract

**Background:**

Breast cancer (BCa)-related mortality still remains the second leading cause of cancer-related deaths worldwide. Patients with BCa have increasingly shown resistance and high toxicity to current chemotherapeutic drugs for which identification of novel targeted therapies are required.

**Methods:**

To determine the effect of PDBD on BCa cells, estrogen-receptor positive (ER^+^)-MCF-7 and estrogen-receptor negative (ER^-^)-MDA 231 cells were treated with PDBD and the cell viability, apoptotic, cell cycle, Western blot and Promoter assays were performed.

**Results:**

PDBD inhibits cell viability of ER^+ ^and ER^- ^BCa cells by inducing apoptosis without causing significant toxicity in normal breast epithelial cells. While dissecting the mechanism of action of PDBD on BCa, we found that PDBD inhibits Akt signaling and its downstream targets such as NF-κB activation, IAP proteins and Bcl-2 expression. On the other hand, activation of JNK/p38 MAPK-mediated pro-apoptotic signaling was observed in both ER^+ ^and ER^- ^BCa cells.

**Conclusion:**

These findings suggest that PDBD may have wide therapeutic application in the treatment of BCa.

## Background

Patients with estrogen receptor-negative breast cancer (ER^- ^BCa) have a median survival of 10–12 months when compared to patients with for ER-positive (ER^+^) BCa who have a median survival of 40–48 months [1]. Limited effectiveness of current chemotherapeutic drugs such as tamoxifen, paclitaxel and docetaxel, shows severe side effects in the BCa patients [2], these realities underscore the importance of identifying novel targeted therapies with minimal side effects to treat this deadly disease.

Akt plays a major role in the regulation of cell survival, apoptosis, and oncogenesis [3]. Activation of Akt negatively regulates the programmed cell death signaling either by blocking or inhibiting the pro-apoptotic proteins such as BAD, Forkhead transcription factors and GSK-3β [3-5]. The observations from cell culture studies suggests that activation of Akt leads to the phosphorylation of IKK which in turn results in NF-κB activation and cell survival [6]. Akt regulates cell cycle by phosphorylating the cell cycle inhibitors p21 and p27 resulting in uncontrolled cell proliferation in various cell types [7-9]. Additionally, Akt increases cyclin D1 expression thereby aiding cancer cell growth and proliferation [10].

Mitogen activated protein kinases (MAPKs) which are serine/threonine protein kinases involved in carcinogenesis due to their ability to stimulate cell proliferation and survival [11]. Three major subfamilies have been described: extracellular-regulated kinases (ERK), c-Jun N-terminal kinase (JNK), and p38 kinase; depending on the cellular context and stimulators these signaling pathways will be activated following phosphorylation of downstream events which will decide the fate of the cell. In the MAPK pathway, the small G-protein, Ras activates Raf-1 which in turn activates MEK-1 resulting in the activation of p44 (ERK1) and p42 (ERK2) which is known to cause cellular proliferation and inhibit apoptosis [12]. However, growing evidence suggesting the dual role of the MEK/ERK pathway in cell survival and apoptosis [13]. On the other hand, JNK and p38 kinases are generally involved in the regulation of pro-apoptotic signaling in many cell types [14].

Current therapeutic modalities for BCa are usually associated with toxicity and side effects thereby indicating novel targeted therapies are much needed. Recently, much attention is being paid to natural compounds and several groups have demonstrated their usefulness either for chemoprevention or chemotherapy on BCa. The lack of mechanistic details about these compounds has impeded bringing them to the main stream of medicine for prevention or treatment of BCa. Naturally occurring polyphenolic antioxidants are recognized as one of the most effective classes of cancer-preventive agents [15-17], because they reduce oxidative stress a known contributor to carcinogenesis with little or no systemic toxicity [18]. Work in our laboratory is focused on dissecting the mechanism of action of natural compounds and more importantly, to discover promising lead components for the development of anti-cancer drugs that specifically target BCa. Recently, we reported that a polyherbal medicine is currently in practice as complementary and alternative therapy for the treatment of BCa in the southern parts of India [19], which were shown to inhibit ER^+ ^and ER^- ^BCa cells in cell culture models [19]. We have isolated an active ingredient, (8'*Z*)-3-pentadec-10-enyl-benzene-1, 2-diol (PDBD) from this polyherbal mixture which has proved to be effective on BCa cells [20]. This study is focused on determining the molecular mechanism of action of PDBD on BCa.

## Methods

### Cell lines

MCF-10A, MDA 231, MCF-7, ZR-75-1, Hs578T and MDA 435 cells were purchased from American Type Culture Collection (Manassas, VA). All the cell lines were grown in DMEM supplemented with 10% fetal bovine serum and 1% L-glutamine.

### Natural compounds and caspase inhibitor

**Figure 1:**
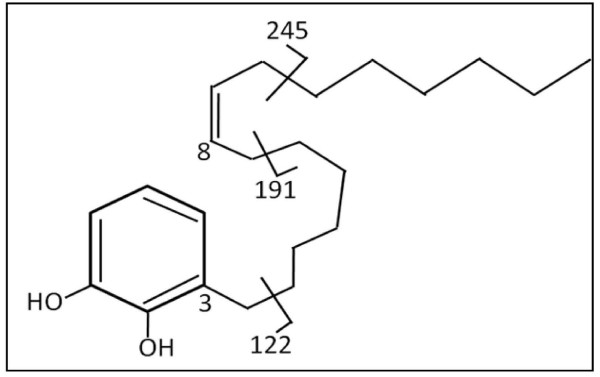
**Structure of [(8'*Z*)-3-pentadec-10-enyl-benzene-1, 2-diol (PDBD)**.

(8'*Z*)-3-pentadec-10-enyl-benzene-1, 2-diol (PDBD) was the major compound isolated from Polyherbal mixture in the Dr. Rohr's laboratory at the University of Kentucky with a purity of 99.5% (Figure 1) [20]. Caspase-3 inhibitor, zDEVD-CHO was purchased from Promega Corporation (Madison, WI).

### Cell viability, Apoptotic assays and Cell Cycle analysis

Cells were treated with PDBD or vehicle (DMSO) for 24 h at different concentrations and cell survival curve was plotted using MTT assay [21]. Also, Annexin V-FITC staining assays and TUNEL assays were performed on five different BCa cells and treated with 4, 6 or 8 μM PDBD followed by flowcytometric analysis as described earlier [22,23]. Cell cycle analysis in MCF-7 and MDA 231 cells was performed following treatment with PDBD (3 μM and 2 μM respectively) using flow cytometric analysis as described earlier [22].

### Western Blot analysis

MDA 231 and MCF-7 cells were treated with PDBD (4 and 6 μM, respectively) for varying time intervals and cell lysates were subjected to Western blot analysis using Akt, ERK, pERK1/2, MEK-1, MEK-4, MEKK-1, pMEK-1/2, pMEK-3/6, Bcl-2, survivin, cdk-2, cdk-4, cdk-6, cyclin D1, cyclin E and NF-κB subunits p50, p55 and p65 from Santa Cruz Biotechnology (Santa Cruz, CA); XIAP and Bcl-xL from MBL International (Woburn, MA) and, pAkt (ser 473), PI3K p85 and Histone H3 from Cell signaling technology (Danvers, MA). β-actin and GAPDH from Santa Cruz Biotechnology was used as the loading control.

### Kinase assay

PI3 kinase assay was also performed to determine the effect of PDBD on PI3K expression and activity. PDBD- and vehicle-treated cells were subjected to PI3 kinase assay using colorimetry as described earlier [24].

### ELISA for IκB-α activity

MDA 231 cells were treated with PDBD for 6 and 12 hours, and IκB-α activity was quantified using IκB-α ELISA kit (Calbiochem, Gibbstown, NJ) as described earlier [24].

### ELISA studies for NF-κB activity

MDA 231 cells were treated with PDBD for 6 and 12 hours, and binding studies were performed to determine the activity of NF-κB p65 subunit using universal EZ-TFA transcription factor assay kit (Millipore, Bedford, MA) as described earlier [25].

### Transient transfection and luciferase assay

MDA 231 cells (at 70–80% confluency) were transiently transfected using Lipofectamine plus reagents from Invitrogen Corporation (Carlsbad, CA) with 4 μg of the NF-κB luciferase construct (containing two tandem repeats of NF-κB-responsive sites) in the presence of a vector containing Renilla luciferase to normalize transfection efficiency as described earlier [26,27]. Transfected cells were either left untreated or treated with PDBD as indicated and the cells were harvested after 24 h to determine NF-κB promoter activity.

### Fluorometry for caspase-3 activation

MDA 231 cells were treated PDBD alone or caspase-3 inhibitor alone or a combination of both for 24 h and caspase-3 activity was determined using the ApoAlert caspase-3 fluorescent assay kit as described earlier [26].

### Statistical analysis

all the experiments were performed three times to ascertain reproducibility of the results and the data shown are representative of three experiments. The student's t test was used to calculate statistical significance.

## Results

### PDBD inhibits cell survival and induces apoptosis on ER^+ ^and ER^- ^BCa

**Figure 2:**
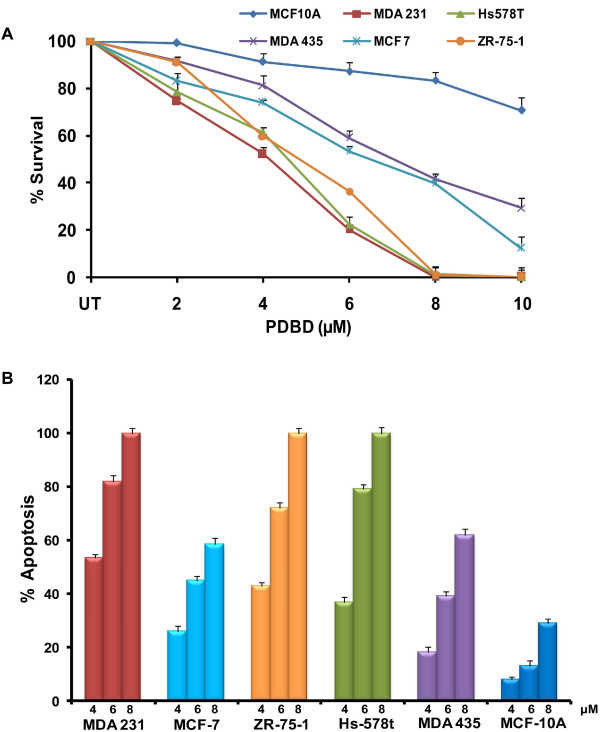
**A. BCa cells were treated with varying concentrations of PDBD for 24 h and MTT assay was performed to determine cell viability**. Each data point represents the mean ± SD for four wells. **B**. PDBD induced apoptosis in BCa cells. Bar graph shows the percentage of apoptosis. The base-line apoptosis in the untreated group was normalized with data in the treated group. The data shown are representative of mean ± SD from three independent experiments.

To determine the anti cancer effect of PDBD, we conducted cell viability and apoptotic assays on a panel of ER^+ ^(MCF-7 and ZR-75-1), ER^- ^(MDA 231, MDA 435 and Hs578T) BCa and normal breast epithelial (MCF-10A) cells. As seen in figure 2A, MDA 231, Hs578t and ZR-75-1 cells were highly sensitive to PDBD when compared to MCF-7 and MDA 435 cells. Interestingly, there was no significant alteration in the viability of normal breast epithelial cell line, MCF-10A suggesting that PDBD selectively targets BCa cells.

To determine whether inhibition of cell viability by PDBD is due to the induction of apoptosis, all the five BCa cell lines (Hs578T, ZR-75-1, MCF-7, MDA 231 and MDA 435) and normal breast epithelial cells (MCF-10A) were subjected to Annexin V-FITC and TUNEL assays. PDBD at 8 μM concentration induced almost 100% apoptosis in MDA 231, Hs578T and ZR-75-1 when compared to 56% and 58% of apoptosis in MCF-7 and MDA 435 cells respectively (Figure 2B). However, PDBD failed to induce significant levels of apoptosis in the normal breast epithelial cell line, MCF-10A (Figure 2B). The apoptotic index was confirmed by Annexin-V-FITC/PI and TUNEL assays.

### Role of PDBD in cell cycle regulation in BCa cells

We investigated whether PDBD plays a role in the regulation of cell cycle and found that PDBD treatment induced a strong G_0_/G_1 _cell cycle arrest observed in time dependent manner. In MDA-231 cells, a G0/G1 phase of cell cycle distribution was 64.5% at 12 h following treatment with PDBD, 66.3% and 75.36% at 24 and 48 hours respectively with a concomitant decrease in the percentage of cells in the S and G_2_/M phase (Table 1). A similar G_0_/G_1 _arrest was observed in MCF-7 cells following treatment with PDBD (Data not shown).

**Table 1:** Effects of PDBD on cell cycle distribution of PDBD.

Cell cycle	Treatments	MDA 231 (in %)		
		
		12 h	24 h	48 h
G0/G1 phase	UT	46.85	36.77	39.53
	
	PDBD	64.53	66.33	75.36

S phase	UT	33.49	56.66	54.71
	
	PDBD	28.14	33.67	15.77

G2/M phase	UT	4.15	6.57	5.76
	
	PDBD	7.34	0.0	8.86

**Figure 3:**
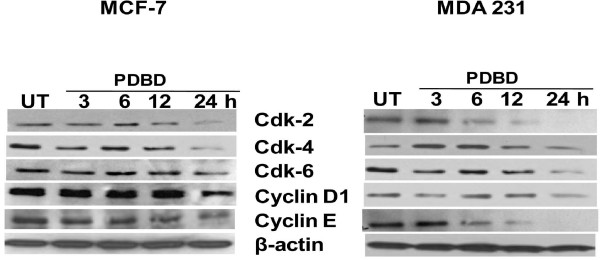
**MCF-7 and MDA 231 cells were treated with PDBD and cell lysates were subjected to Western blot analysis using cdk-2, cdk-4, cdk-6, Cyclin D1 and Cyclin E**. β-actin was used as a loading control.

Next, we examined whether PDBD regulates the expression of G_0_/G_1 _cell cycle proteins in MCF-7 and MDA 231 cells. As seen in figure 3, PDBD downregulated the expression of Cdk-2, Cdk-4 and Cdk-6 in a time dependent fashion in both MCF-7 and MDA 231 cells. In addition, Cyclin E and Cyclin D1 expressions were also downregulated following treatment with PDBD in both BCa cell lines. Collectively these observations suggest that PDBD alters the expression of G_0_/G_1 _regulatory proteins thereby causing cell cycle arrest in BCa cells.

### PDBD inhibits Akt signaling without altering PI3K activity in BCa cells

**Figure 4:**
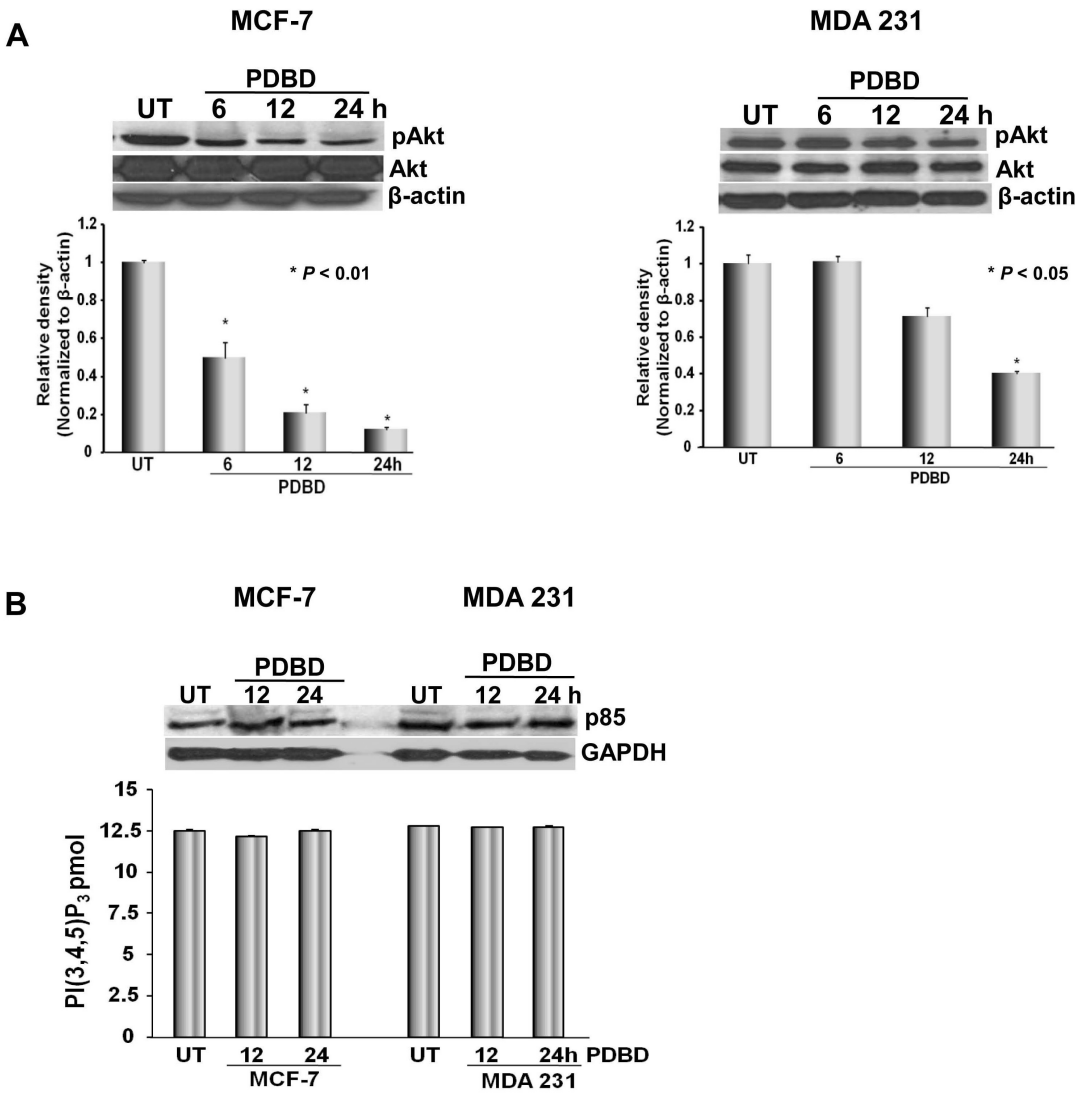
**A. MCF-7 and MDA 231 cells were treated with PDBD and cell lysates were subjected to Western blot analysis (upper panel) using pAkt (ser473) and Akt**. Lower panel are the bar graphs representing the relative density of pAkt in MCF-7 and MDA 231 cells that were normalized with β-actin. **B**. MCF-7 and MDA 231 cell lysates were subjected to Western blot analysis (upper panel) or colorimetric assay (lower panel) using PI3K antibody.

**Figure 5:**
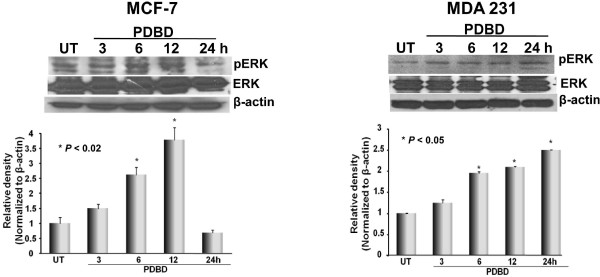
**MCF-7 and MDA 231 cells were treated with PDBD and subjected to Western blot analysis using pERK and ERK**. Lower panel are the bar graphs representing the relative density of pERK in MCF-7 and MDA 231 cells that were normalized with β-actin.

The protein kinase, Akt, functions as a molecular nexus for a number of signaling pathways that regulate cell growth, cell survival, and tumor progression, and its activity has been implicated in the inhibition of apoptosis and promotion of angiogenesis [28]. PDBD inhibited pAkt (ser 473) expression 6 h after treatment in MCF-7 cells, whereas, in MDA-231 cells, inhibition of pAkt expression was seen at 24 h after treatment. No alteration in total Akt levels in MDA 231 cells were seen (Figure 4A). Then, we determined whether PDBD targets the upstream event of Akt, the PI3K and found that no alteration of either the expression (upper panel) or activity (lower panel) of PI3 Kinase in BCa cells (Figure 4B) were seen suggesting that PDBD specifically either Akt or its downstream signaling of BCa. While dissecting the effect of PDBD on Extracellular-regulated kinase (ERK) signaling, we found that gradual upregulation of pERK expression was seen in MCF-7 and MDA 231 cells. Total ERK levels remained constant in both the BCa cell lines following treatment with PDBD for upto 24 h (Figure 5).

### PDBD inhibits NF-κB activation in MDA 231 cells

**Figure 6:**
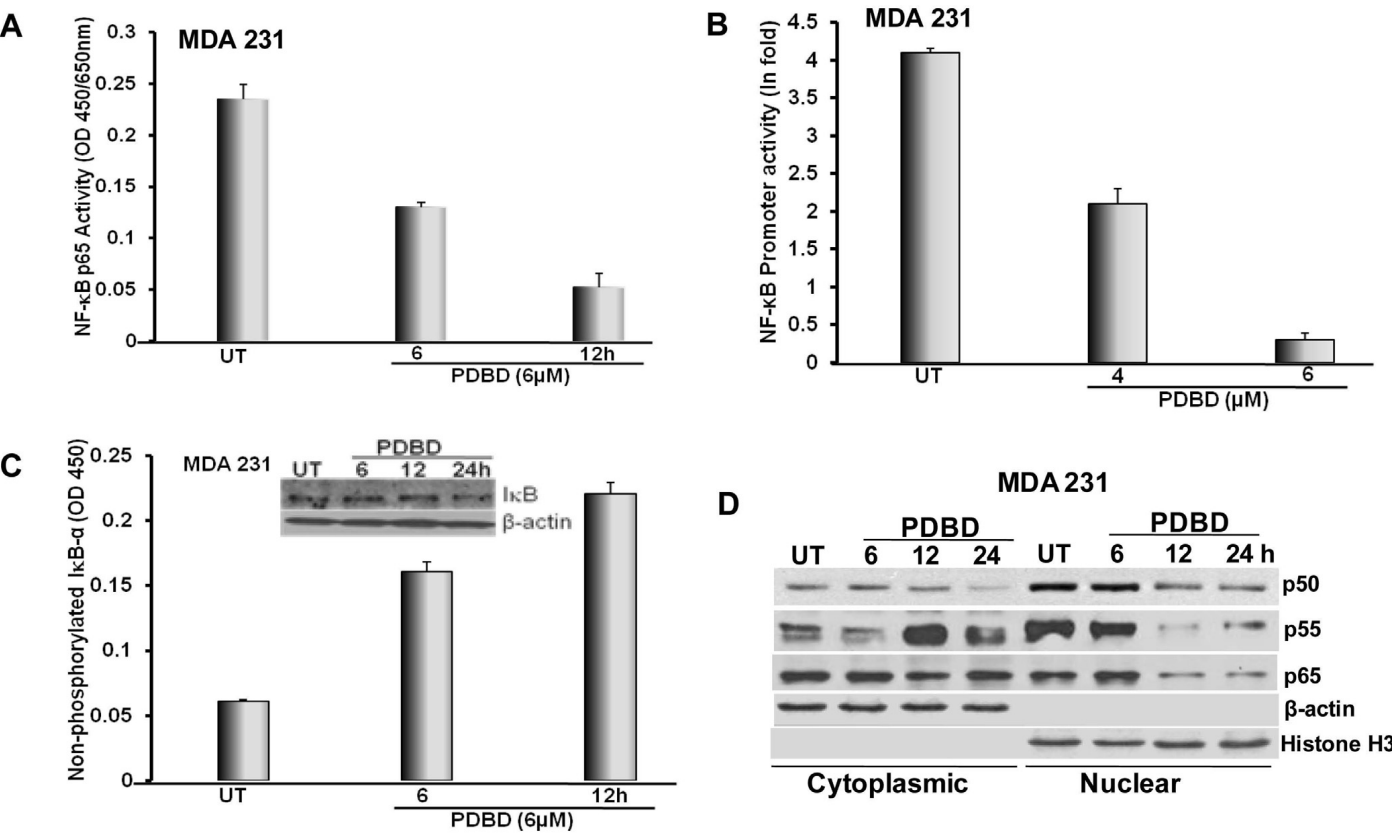
**A. MDA 231 cells treated with PDBD and subjected to NF-κB binding assay and NF-κB p65 activity was studied to determine DNA binding activity**. **B**. MDA 231 cells were transiently transfected with NF-κB-luc, treated with varying concentrations of PDBD for 24 h and reporter studies were performed to determine NF-κB promoter activity. pRL-TK was used as the rennilla control plasmid. **C**. MDA 231 cells were treated with PDBD and lysates were subjected to either Western blot analysis (upper panel) or ELISA (lower panel) for expression profile of non-phosphorylated IκB-α. **D**. Nuclear and cytoplasmic fractions were obtained from MDA-231 cells, which were subjected to Western blot analysis using NF-κB p50, p55 and p65. β-actin was used as the loading control for cytoplasmic fraction and Histone H3 was used as the loading control for nuclear fraction.

Since there was no significant downregulation of pAkt expression in MDA 231, we investigate whether the downstream events of Akt signaling are affected by PDBD. Nuclear factor-κB is a transcription factor which is involved in cell survival and proliferation and has been established as one of the major downstream targets of Akt [29]. PDBD down regulated NF-κB p65 activity (Figure 6A) and also inhibited NF-κB at the promoter level (Figure 6B) in MDA 231 cells. Then, we analyzed IκB status, and our results suggest that PDBD is capable of maintaining IκB-α in the non-phosphorylated form thereby inhibiting the nuclear translocation of the active NF-κB subunits (Figure 6C). Also, as expected, a significant down regulation of NF-κB subunits (p65, p55 and p50) in a timely manner in the nuclear fraction suggesting that PDBD effectively inhibits NF-κB activation (Figure 6D).

### PDBD regulates Pro-Apoptotic and Pro-Survival Proteins in BCa

**Figure 7:**
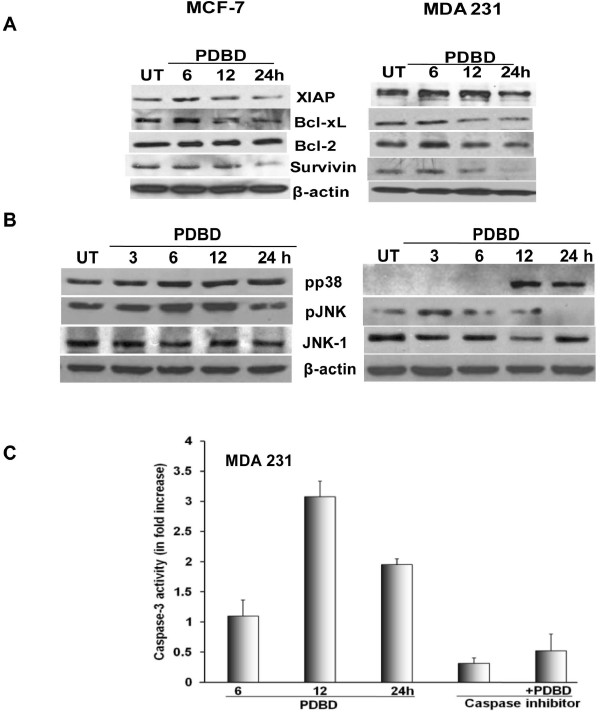
**A. MDA 231 cells treated with PDBD and cell lysates were subjected to Western blot analysis using XIAP, Bcl-xL, Bcl-2 and survivin**. **B**. Western blot analysis using pp38, pJNK, JNK-1 expression in MCF-7 and MDA 231 cells. **C**. MDA 231 cells were treated with PDBD alone, caspase inhibitor alone or a combination of PDBD and caspase inhibitor for varying time intervals and were subjected to caspase-3 activation studies using flourometric assay.

The Bcl-2 family of proteins and IAPs are a well established class of proteins that are involved in the regulation of cell survival and apoptosis [30]. In our results, PDBD downregulates the expression of XIAP, Bcl-2, Bcl-xL and survivin at 12 h onwards in MDA 231 cells, however, in MCF-7 cells there were no changes in the expression levels of Bcl-2, but survivin, Bcl-xL and XIAP were down-regulated following treatment with PDBD at 24 h (Figure 7A). The JNK and p38 MAP kinase pathway proteins are associated with pro-survival as well as pro-apoptotic functions [31] and therefore the expression levels of pJNK and pp38 were investigated. The up regulation of pJNK were found from 3 h onwards in MCF-7 and MDA 231 cells, however, pp38 was upregulated at 3 h in MCF-7 cells and at 12 h MDA 231 cells (Figure 7B). Also, PDBD induced caspase-3 activation (~3.1-fold) in MDA 231 cells (Figure 7C) suggesting that PDBD induces JNK- and p38-mediated pro-apoptotic signaling in BCa.

## Discussion

Discovery of active compounds from natural products have gained enormous importance in the field of BCa therapy. In the present study, we have identified a potent compound PDBD from a polyherbal mixture which specifically targets BCa cells without causing adverse effects on normal breast epithelial cells. Interestingly, our results suggest that ZR-75-1 cells are more sensitive when compared to MCF-7 cells, which may be due to the absence of caspase-3 in MCF-7 cells. The difference in PDBD sensitivity in MDA 231 and MDA 435 cells may be due to the over expression of Erb-B2 in MDA 435, which regulates cell survival and proliferation in several cancer types including BCa [32-35].

Dysregulation of the expression of the cyclins and cdks are involved in cell cycle, and which has been found to be a hallmark in various types of cancer. Cyclin D1, a component subunit of Cdk-4 and Cdk-6, is a rate-limiting factor in progression of cells through the first gap (G_1_) phase of the cell cycle [36]. Downregulation of Cdk-2, Cdk-4, Cdk-6, Cyclin E, Cyclin D1 expression by PDBD suggests that it targets several cell cycle regulatory proteins in BCa cells.

The serine/threonine protein kinase, Akt, plays critical role in mammalian cell survival and has been shown to be activated in various cancers including BCa [37,38]. Some clinical studies suggested that activation of Akt correlates with HER-2 expression and these patients tend to have higher rate of relapse to tamoxifen therapy [39-42]. Recent studies report 15%–30% of BCa patient's express high pAkt levels which was associated with resistance to chemotherapeutic agents. The ability of PDBD to inhibit pAkt expression in our study suggests that either PDBD alone or a combination of PDBD with other chemotherapeutic agents like tamoxifen and doxorubicin may enhance the therapeutic potential of current chemotherapy drugs [43,44]. While dissecting the involvement of PI3K-mediated Akt signaling, we found that PDBD fails to alter the expression and kinase activity of PI3K in MCF-7 and MDA 231 cells. Though, MDA 231 cells were more sensitive to PDBD when compared to MCF-7 cells, pAkt expression was not significantly downregulated in MDA 231 cells suggesting that PDBD directly targets the downstream events of PI3K/Akt signaling in MDA 231 cells.

NF-κB activation regulates cell survival [45,46] and it also simultaneously inhibits the expression of several pro-apoptotic proteins in various cell types [3,47]. In our studies we found that PDBD inhibited phosphorylation of Akt in MCF-7 cells when compared to MDA 231 cells; so we intended to determine whether PDBD regulates Akt downstream events which may possibly result in inhibition of cell survival. Several lines of evidence suggest a constitutive overexpression of NF-κB in ER^- ^BCa compared to ER^+ ^BCa [48-50]. The p65 subunit of NF-κB is overexpressed in most of the BCa cell lines and in BCa tumor tissues [48]. Interestingly a higher percentage of NF-κB activation (98.7%) was found in human BCa tissues from Taiwanese women which was correlated with higher percentage of patients with metastatic BCa [51]. In our study, PDBD inhibited NF-κB activation at the promoter and protein level in MDA 231 cells suggesting that PDBD can be used as a potential therapeutic agent for BCa. Several natural compounds have the ability to downregulate NF-κB activation and some of the compounds that we investigated in our laboratory namely curcumin [22], Withaferin A [26] and Psoralidin [24], also shown the ability to downregulate the NF-κB activation in many cancer types.

Increased expression of phosphorylated JNK and p38 by PDBD in both MCF-7 and MDA 231 cells suggest that activated JNK and p38 play a role in the induction of apoptosis in BCa cells [52]. Descriptively, the inhibition of Akt pathway and the simultaneous activation of p38/JNK pathway may attribute to the anti cancer activity of PDBD in BCa cells. It is important to investigate whether inhibition of cell proliferation and induction of apoptosis by PDBD is associated with the down-regulation of pro-survival signaling. Our results suggest that PDBD downregulates the expression of XIAP, Bcl-xL and surviving which might lead to chemosensitization of BCa cells.

## Conclusion

PDBD inhibits pro-survival signaling such as Akt, MEK and NF-κB with a simultaneous induction of pro-apoptotic proteins in the BCa cells resulting in inhibition of cell survival and proliferation. Additionally, PDBD causes a G_0_/G_1 _cell cycle arrest in both MDA 231 and MCF-7 cells which is also an important aspect for the treatment of cancer. Collectively our results suggest that further investigation of PDBD *in vivo *models may help bring this potent molecule into the main stream of medicine for the treatment of BCa.

## Abbreviations

BCa: breast cancer; ER: estrogen receptor; PDBD: (8'*Z*)-3-pentadec-10-enyl-benzene-1, 2-diol; IAP: inhibitors of apoptosis protein; MAPK: Mitogen activated protein kinase; ERK: extracellular-regulated kinase; JNK: c-Jun N-terminal kinase; PI3K: Phosphotidyl Inositide-3-Kinase.

## Competing interests

The authors declare that they have no competing interests.

## Authors' contributions

SK, SS, RK and RG performed experiments. JR and CD designed the experiments, prepare the manuscript and supervised the project. All authors read and approved the final manuscript.

## Pre-publication history

The pre-publication history for this paper can be accessed here:

http://www.biomedcentral.com/1471-2407/9/41/prepub
